# Shape (but not volume) changes in the thalami in Parkinson disease

**DOI:** 10.1186/1471-2377-8-8

**Published:** 2008-04-16

**Authors:** Martin J McKeown, Ashish Uthama, Rafeef Abugharbieh, Samantha Palmer, Mechelle Lewis, Xuemei Huang

**Affiliations:** 1 Pacific Parkinson's Research Center, University of British Columbia, Vancouver, Canada; 2 Brain Research Centre, University of British Columbia, Vancouver, Canada; 3 Department of Medicine (Neurology), University of British Columbia, Vancouver, Canada; 4 Biomedical Signal and Image Computing Lab, Department of Electrical and Computer Engineering, University of British Columbia, Vancouver, Canada; 5 Department of Neurology, University of North Carolina, Chapel Hill, NC, USA

## Abstract

**Background:**

Recent pathological studies have suggested that thalamic degeneration may represent a site of non-dopaminergic degeneration in Parkinson's Disease (PD). Our objective was to determine if changes in the thalami could be non-invasively detected in structural MRI images obtained from subjects with Parkinson disease (PD), compared to age-matched controls.

**Results:**

No significant differences in volume were detected in the thalami between eighteen normal subjects and eighteen PD subjects groups. However significant (p < 0.03) shape differences were detected between the Left vs. Right thalami in PD, between the left thalami in PD and controls, and between the right thalami in PD and controls using a recently-developed, spherical harmonic-based representation.

**Conclusion:**

Systematic changes in thalamic shape can be non-invasively assessed in PD in vivo. Shape changes, in addition to volume changes, may represent a new avenue to assess the progress of neurodegenerative processes. Although not directly discernable at the resolution of standard MRI, previous pathological studies would suggest that the shape changes detected in this study represent degeneration in the centre median-parafascicular (CM-Pf) complex, an area known to represent selective non-dopaminergic degeneration in PD.

## Background

The thalamic changes seen in Parkinson Disease (PD) may represent selective non-dopaminergic degeneration [1], as there is selective neuronal loss in the centre median-parafascicular (CM-Pf) complex in Parkinson's disease [2], yet relative preservation of neurons in the limbic (mediodorsal and anterior principal) thalamic nuclei. Henderson et al. examined the thalamic intranuclear nuclei in 10 normal controls and 9 patients with PD [3]. As expected, they found *α*-synuclein-positive Lewy bodies in these nuclei in the thalamus, but they also found a significant reduction (40–55%) in the total neuronal number in the caudal intralaminar (CM-Pf) nuclei, regions that receive glutaminergic innervation [3]. This contrasted with the 70% loss of pigmented nigral neurons. A factor analysis has demonstrated that the size of neurons in the motor cortex is negatively correlated with the size and number of neurons in its thalamic relay, the VLp. There is also a positive correlation between the number of ventral anterior (VA) neurons and the pre-supplementary motor area (SMA) [4].

Bacci et al. suggested that CM-Pf degeneration may partially counteract the consequences of dopamine neuronal loss, as thalamic and dopamine inputs have antagonistic influence on neurotransmitter-related gene expression [1]. Moreover, the CM-Pf degeneration may be a direct consequence of nigrostriatal denervation, as depleting the striatum of dopamine results in the remaining Pf neurons being particularly hyperactive [5].

The normal role of the CM-Pf complex is incompletely understood, although it is clearly related to basal ganglia function [6]. The Pf nuclei receive input from the spinal cord and project to the striatum [7]. These projections may carry specific temporally-patterned inputs to striatal targets [8]. While the CM-Pf complex has traditionally been considered part of the reticulo-thalamo-cortical activating system, a recent proposal suggests that the CM-Pf complex participates in sensory driven attentional processes, particularly unexpected events [9].

The advent of modern imaging techniques has allowed the noninvasive *in vivo *assessment of brain structures, such as the thalamus, in disease states. Thalamic morphological changes have been detected chronically after cortical injury, such as middle cerebral artery (MCA) infarction after 1 yr [10,11] and tumor resection after ~2 yrs [12]. The study by Hulshoff Pol [12] detected a 5% decrease in ipsilateral thalamus and, interestingly, a 4.5% increase in contralateral thalamic volume after unilateral tumor resection, presumably on the basis of a compensatory mechanism.

In PD and related disorders, some studies of structural and functional imaging have detected thalamic changes. Thalamic grey matter changes have been detected contralateral to unilateral Parkinsonian resting tremor [13]. In PD with dementia, the thalamus, in addition to the hippocampus and anterior cingulate, represent the regions most affected [14]. Functionally, there is a connection between a major component in F-DOPA uptake in the striatum and a component from fluoro-deoxy – glucose (FDG), which had positive loadings in the thalamus and the cerebellum [15].

Most morphological studies based on imaging involve a number of steps manipulating the brain images. A typical approach would be to warp ("spatially normalize") the brain images to a common space [13]. Further smoothing of the data (e.g. using an isotropic 12 mm Gaussian kernel) to minimize the effects of misregistration between different normalized brains may affect the ability to make inferences about small, subcortical structures like the thalamus. In fact this "Voxel Based Morphometry" approach has thus been a controversial approach (e.g., see discussions in [16] and [17]).

Recent approaches try to reduce errors due to misregistration by aligning the subjects at the region of interest (ROI) level, as opposed to the whole brain level [18-20]. However, these approaches are designed to deal with a different problem, namely that of summarizing fMRI activation from several subjects. To quantify differences in morphology, it would be necessary to examine the different transformations required to warp each subject's ROIs to the examplar ROI shape – a non-trivial task.

An alternative approach to warping brains to the same space is to segment brain structures individually on unmanipulated (i.e. unregistered and unwarped) brains [21]. Because no registration of the brain images is done, this requires summarizing the individual brain structures in a way that they are invariant to positioning of the head in the scanner. For example, simply estimating the volume of an ROI such as the thalamus has this property, as it is invariant to the individual coordinate frame used. A number of such invariants (e.g. spatial variance) have been proposed to summarize the shape of brain structures [22], or even characterize the distribution (texture) of activation maps in fMRI [23].

**Figure 1:**
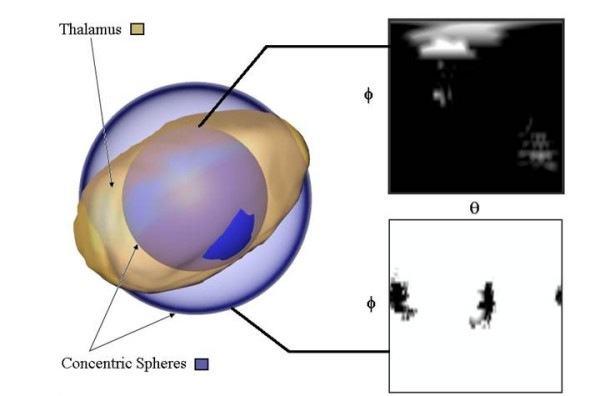
**The SPHARM-based method for shape determination**. The shape to be specified (the thalamus) and two concentric spherical shells are shown. On the right is the intersection between the thalamus and shells as a function of rotation (*θ*) and azimuth (□). The rotation angle spans from 0 to 2*π *radians, and the azimuth angle is from 0 to *π *radians.

We have recently proposed a method based on spherical harmonics (SPHARM) which provided a unique representation of brain structures, including regions with possible topological disconnections, such as the lateral ventricles [24]. In brief, the method involves first finding a spherical shell which encompasses the entire ROI. Subsequent smaller concentric shells are then derived and the intersection between the progressively smaller spherical shells and the brain structure is computed (Figure 1). The results from the intersections are then combined into a unique feature vector containing approximately 100's or 1000's of elements. This feature vector provides a unique representation of the shape which is independent of the spatial orientation of the structure (see Methods).

We examined the thalami from 18 PD subjects and 18 age-matched controls. Using the above technique, we found significant differences between the two groups in the shape of the thalami, but not in the volume. This suggests that significant thalamic changes can be assessed noninvasively in PD, suitable for longitudinal studies.

## Results

There were no significant differences in volume between sides in either controls or PD subjects, nor between controls and PD subjects in either the left or right thalamus (Table 1). In contrast, the SPHARM-based method found significant shape differences between the left and right thalamus in PD but not in controls. Significant shape differences between PD and controls were detected in both the left and right thalami.

**Table 1:** Results of volumetric and shape analysis. Numbers indicate the p-values obtained from the permutation test.

**Group**	**Volume**	**SPHARM**
Control, Left vs. Right	0.5630	0.1470
PD, Left vs. Right	0.5780	0.0060
Left thalamus, PD vs. Control	0.4150	0.0270
Right thalamus, PD vs. Control	0.1730	0.0290

For PD subjects, an ANOVA examining the influences on the distance metric (Eqn 8) of acquisition site, side of symptoms or duration (F(1,20) = 1.9, p = 0.18; F(1,20) = 1.59, p = 0.22; F(17,20) = 1.35, p = 0.26; respectively). As all PD subjects were right handed, handedness could not be tested in this group. Similarly, an ANOVA on the distance metric for all subjects examining site of acquisition (F(1,72) = 0.14, p = 0.71) or handedness (F(1,72) = 0.16, p = 0.69) did not find factors of significance with a fixed effects model.

**Figure 2:**
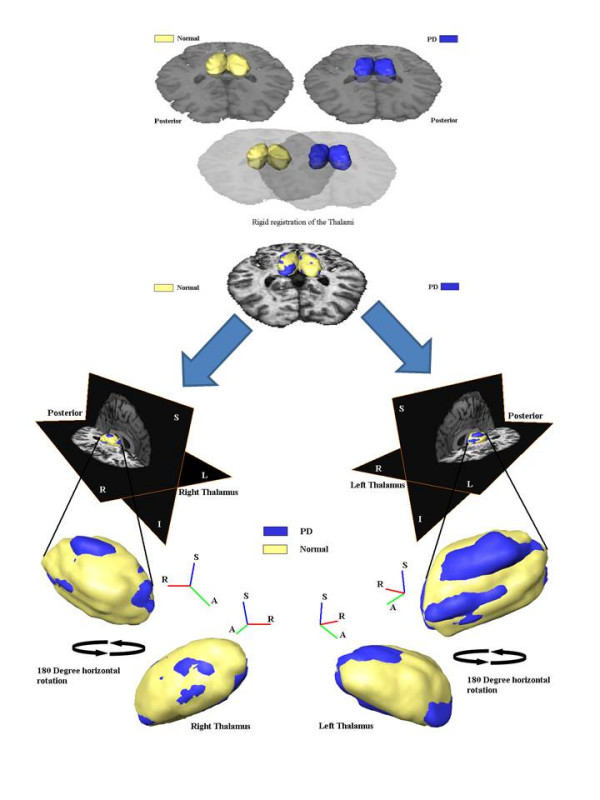
**Two sets typical thalami registered and shown on brain from a PD subject**. Note that the registration here is solely for visualization purposes, and is not required for the calculation of shape differences. Also, although the thalami here were first smoothed with a 12 mm FWHM Gaussian kernel for visualization purposes, no smoothing was performed for the shape analysis and group comparison.

In order to better visualize and intuitively assess the shape differences in thalami between PD subjects and controls, we took thalami that had "typical" feature vectors (i.e. feature vectors closest to the mean of each group) and assumed that they represented exemplar shapes. We then spatially aligned these exemplar shapes (Figure 2). There appeared to be greater differences in the left thalami between controls and PD subjects. The largest differences appeared to be along the dorsal surface. Note that the registration of the thalami in this instance was solely for visualization purposes and was not incorporated into the analysis.

## Discussion

It is well known that changes in the thalamus can be seen chronically after cortical injury [25,26]. This is not only due to direct effects of axonal damage, as axonal-sparing cortical lesions also result in thalamic degeneration [27]. Such thalamic degeneration probably involves both anterograde and retrograde processes [28] and may be mitigated by growth factors [29,30]. Brain development has a critical role on the extent of thalamic changes after a cortical lesion. Animal models have determined that perinatal lesions are far less likely to induce thalamic changes, compared to when the cortical lesions are made prenatally [31] or in adulthood [32]. In contrast to the secondary effects of thalamic changes from cortical lesions, the thalamic changes in PD are related to selective non-dopaminergic neurodegeneration [1].

Consistent with prior results, we found no significant differences in the volume of the thalami between PD subjects and controls [3]. However, for the first time, we have demonstrated that the *shape *of the thalami undergoes systematic changes in PD. The reason that shape may change but not the volume may be due to the fact that particular nuclei (e.g CM-Pf) are involved, and thus, at the typical resolution of MRI, this does not result in significant changes in the overall volume. Alternately, since thalamic volume may actually increase as a compensatory mechanism [12], other regions of the thalamus may hypertrophy.

The ability to non-invasively quantify subtle morphological shape changes appears to be a powerful technique. We utilized standard structural MR imaging techniques without any special sequences nor any special scanner resolution requirements. We obtained robust results from pooling data from two different centres using two different types of scanners.

We used manual segmentation of the thalami in this paper. Automatic segmentation of subcortical structures is an area of ongoing research [33], and often requires the tuning of many parameters, especially when the images may be pooled from scanners from different centres. Since the person at each centre doing the segmentation was blinded to disease status, it would be unexpected that a systematic bias was introduced into our results. Even then, any misspecification of the same ROI across subjects would tend to increase inter-subject variability and presumably reduce discriminability across groups making the task harder for the shape analysis approach.

We used SPHARM-based invariant descriptors to quantify the shape of the thalami. The main advantages of such a method is that it does not require that all brain images be warped to a common space, nor does it require that the brain images be aligned in any way. A drawback of these invariant features approach is that it is difficult to invert the feature vectors, i.e. once given all the values of the different invariants, it is impossible to reconstruct the original image which gave that feature vector – analogous to the fact that given the volume of an object, it is impossible to uniquely reconstruct the original shape. We therefore cannot create a "typical" brain structure by averaging the feature vectors and create the image that would give this feature vector (e.g. to create an "average left thalamus"). However, it is possible to cluster structures in the feature space and find the brain structure whose feature vector is in the middle of the cluster so as to use it as an exemplar shape, which we have done (Figure 2).

It is difficult to determine if the shape differences we detected are attributable to any specific nuclei. However, based on prior pathological studies, it would be likely that the differences we detected were related to degeneration in the CM-Pf complex. Given that progressive supranuclear palsy (PSP) has even greater involvement of VLp than PD [4], it remains to be seen if thalamic shape is a discriminable feature between these two conditions.

We did not detect any association between overall shape change and handedness, and dominant side presentations or presence/absence of tremor. This may be due to the relatively small sample size employed in this study. However, because the feature vectors consist of many different components, we don't discount that there may be a subset of components that are sensitive to these disease parameters.

## Conclusion

Our results suggest that systematic changes in thalamic shape can be non-invasively assessed in PD in vivo and that shape changes, in addition to volume changes, may represent a new avenue to assess the progress of neurodegenerative processes. Although we cannot state which parts of the thalamus are directly affected, previous pathological studies would suggest that the shape changes detected in this study represent degeneration in the centre median-parafascicular (CM-Pf) complex, an area known to represent selective non-dopaminergic degeneration in PD.

## Methods

The study was approved by the appropriate Institutional Review Boards and Ethics Boards of the University of British Columbia (UBC) and the University of North Carolina (UNC). All structural data were obtained as part of fMRI studies whose results are reported elsewhere (e.g., [34]).

### MR Imaging at the University of British Columbia

All subjects gave written informed consent prior to participating. Nine volunteers with clinically diagnosed PD participated in the study (5 men, 4 women, mean age 68.1 ± 6.8 years, 7 right-handed, 2 left-handed). All subjects had mild to moderate PD (Hoehn and Yahr stage 2–3) [35] with mean symptom duration of 3.6 ± 2.6 years. We recruited ten healthy, age-matched control subjects without active neurological disorders (3 men, 7 women, mean age 55 ± 12.4 years, 9 right-handed, 1 left-handed). Exclusion criteria included atypical Parkinsonism, presence of other neurological or psychiatric conditions and use of antidepressants, sleeping tablets, or dopamine blocking agents.

MRI was conducted on a Philips Achieva 3.0 T scanner (Philips, Best, The Netherlands) equipped with a 6 channel Sense head-coil. A high resolution, three dimensional (3D) SPGR image data set of the whole brain consisting of 170 axial slices at a FOV of 256 × 200 mm^2 ^was acquired for WM/GM segmentation purposes and as anatomical reference (inversion prepared 3D T1TFE, TR = 7.746 ms, TE = 3.55 ms, inversion delay = 880 ms, flip angle = 8.00°, voxel dimensions 1.0 × 1.0 × 1.0 mm^3^).

The thalami were one of eighteen specific regions of interest (ROIs) that were manually drawn on each unwarped, aligned structural scan using the Amira software (Mercury Computer Systems, San Diego, USA). Although the thalami were manually segmented on the axial slices, they were carefully examined in the coronal and saggital planes to ensure accuracy. The trained technician performing the segmentation was blinded to the disease state.

### MR Imaging at the University of North Carolina

All subjects gave written informed consent prior to participating. Nine volunteers with clinically diagnosed mild to moderate PD (Hoehn and Yahr stage 2–3 – mean symptom duration of 2.1 ± 2.0 years) participated in the study (5 men, 4 women, mean age 58 ± 12 yrs, all right-handed). We also recruited eight healthy, age-matched control subjects without active neurological disorders (5 men, 3 women, mean age 49 ± 14 yrs, 8 right-handed). Images were acquired on a 3.0 Tesla Siemens scanner (Siemens, Erlangen, Germany) with a birdcage-type standard quadrature head coil and an advanced nuclear magnetic resonance echoplanar system. The head was positioned along the canthomeatal line. Foam padding was used to limit head motion. High-resolution T1 weighted anatomical images were acquired (3D SPGR, TR = 14 ms, TE = 7.7 ms, flip angle = 25°, voxel dimensions 1.0 × 1.0 × 1.0 mm, 176 × 256 voxels, 160 slices).

ROIs (including thalami) were drawn manually by the same trained research associate with assistance from multiple on-line and published atlases (e.g. [36]).

### Thalami Shape Analysis

As described in the technical appendix, the analysis of each shape results in a unique feature vector, of length *n *= 1440. The left and right thalami were analyzed separately.

For comparison, we examined for any differences in volume. The volume of each thalamus was estimated as the number of voxels that each ROI contained multiplied by the volume of a single voxel.

To assess the significance of group differences between feature vectors, we used a permutation test to generate a null distribution of Euclidean distances between feature vectors. The permutation test does not require a priori assumptions about the data distribution, and is thus preferred over T-test and F-test [37]. We assessed the differences in left vs. right thalami in controls, left vs. right in PD subjects, PD vs. controls for the left thalamus, and PD vs. controls for the right thalamus. Although the boundaries of the thalami were determined by visual inspection, in prior work we compared feature vectors derived from thalami segmented from structural scans obtained before and after giving L-dopa medication (as part of another fMRI study) [38]. As expected, no significant differences could be detected in the two groups, suggesting that independent manual segmentation did not incur significant systematic errors.

### Shape Analysis – technical aspects

Let Ψ(*θ*, *φ*) be a function defined on the unit spherewith *θ *and *φ *as the zenithal and azimuthal angles, respectively. The SPHARM representation for this function is given by (1) where $Y_{lm}^{*}\left(\theta ,\varphi \right)$ is the complex conjugate of the *m*^th ^order spherical harmonic of degree *l*. *l *ranges from 0 to *L *[16]. Increasing the value of *L*, also called the bandwidth, improves the representation accuracy at the cost of higher computation time. This definition can also be extended to real valued 3D distributions Ψ(*r*, *θ*, *φ*) (2), where *r *is the distance from the origin to a given voxel. *k *is an index introduced to account for possible degeneracy due to the additional dimension [39].

(1) $$c_{l}^{m}=\int_{0}^{2\pi }d\varphi \int_{0}^{\pi }Y_{lm}^{*}(\theta ,\varphi )\Psi (\theta ,\varphi )\sin(\theta )d\theta$$

(2) $$c_{kl}^{m}=\int_{0}^{\infty }r^{2}dr\int_{0}^{2\pi }d\varphi \int_{0}^{\pi }\sqrt{2}\frac{\sin(\pi kr)}{r}Y_{lm}^{*}(\theta ,\varphi )\Psi (r,\theta ,\varphi )\sin(\theta )d\theta$$

As explained later in this section, rotationally invariant features can be derived from this spherical harmonic representation. In our application, we also need the features to be invariant to any translation of the entire ROI in 3D space. To achieve this, we move the origin of the function Ψ(*r*, *θ*, *φ*), to the centroid of Ψ_*s*_(*r*, *θ*, *φ*), where Ψ_*s*_(*r*, *θ*, *φ*) is given by (3).

(3) $$\Psi_{s}\left(r,\theta ,\varphi \right)=\{\begin{array}{lll} 1 & if & \Psi \left(r,\theta ,\varphi \right)\neq 0\\ 0 & if & \Psi \left(r,\theta ,\varphi \right)=0 \end{array}$$

Since direct computation of (2) is highly inefficient [40], we use an alternate approach by representing the data as a set of spherical functions obtained by intersecting the 3D data with spherical shells. Alternatively, for each value of *r*, Ψ(*r*, *θ*, *φ*) can be visualized as a spherical shell comprising the function values at a distance *r *from the origin. *r *can then be incremented in steps of *t *to encompass the entire ROI. If the initial representation of the function is in the form of a cubic grid (regular isotropic voxels in our case), volumetric interpolation is required to resample the ROI in the spherical coordinate space.

When analyzing multiple subjects' ROIs simultaneously, we define the maximum radius, *R*_*max*_, as the minimum radial distance in voxel count that encompasses all non-zero values of all subjects' ROIs being analyzed. To represent the values from the cubic grid of all ROIs with sufficient accuracy, *2R*_*max *_shells are used. To achieve scale invariance, the shells must be distributed evenly throughout the spatial extent of each ROI. Since the ROI size across subjects is not uniform, shell spacing *t *must be adjusted for each subject separately. This procedure ensures that each shell captures similar features from the 3D ROI irrespective of its scale.

Surface sampling along each of these shells is performed on an equiangular spherical grid of dimensions *2L *× *2L *[40]. The common bandwidth *L *for all shells of all functions is chosen to satisfy the sampling criterion for the largest shell in this set of ROI, namely the one with radius *R*_*max*_. The surface area for this shell represents the maximum surface shell area that needs to be sampled by the equiangular grid; hence, any value of *L *satisfying the required equiangular sampling (*2L *× *2L*) at this shell will be sufficient to represent data from smaller radii shells. The minimum value for *L *is obtained by equating the surface area of this largest shell to the equiangular sampling grid (4). Higher values of L are not used, since it increase computation time with no added benefit. Also, this will result in longer feature vectors, complicating the analysis. Furthermore, when the represented object is a discrete array, higher values of *l*, resulting from a larger *L*, may correspond to sampling noise [39]. Recognizing that in applications pertaining to discrimination, high accuracy in the SPHARM representation is not a necessity, we chose to use the minimum value for *L *as that obtained by (4).

(4) $$4\pi R_{\max}^{2}=2L\times 2L,L=R_{\max}\sqrt{\pi }$$

To obtain the SPHARM representation for all shells, a discrete SPHARM transform is performed at each value of *r *to obtain $c_{rl}^{m}$ (5). Features derived from this representation, however, do not provide a unique function representation [41,42]. For instance, rotating the inner and outer shells by different amount will result in different spatial distribution of function values. However, in this approach, the derived features are insensitive to these rotational transforms, thus resulting in the same feature values for dissimilar spatial distributions.

(5) $$\begin{array}{c} c_{rl}^{m}=\int_{0}^{2\pi }d\varphi \int_{0}^{\pi }Y_{lm}^{*}(\theta ,\varphi )\Psi (r,\theta ,\varphi )\sin(\theta )d\theta \\ r=[1,2,3,...,2R_{\max}] \end{array}$$

Burel and Henocq's original equation (2) does not have this problem, since a part of the basis function is a function of *r*. However, since (2) is computationally intractable [17], we proposed an efficient approach that uses a radial transform (6), derived from (2), to obtain a unique function representation. The transform (6) retains the relative orientation information of the shells, thus the features derived will be sensitive to independent rotations of the different shells, thereby ensuring that *unique *feature representation is obtained.

(6) $$\begin{array}{c} c_{kl}^{m}=\sum_{r=1}^{2R_{\max}}r^{2}\sqrt{2}\frac{\sin(\pi kr)}{r}c_{rl}^{m}\\ k=[1,2,3,...,2R_{\max}] \end{array}$$

The range of *k *could be changed to obtain different lengths of the final feature vector. However, to avoid unnecessarily increasing the feature vector length or losing any important information caused by reducing the range, we choose to keep the range of *k *the same as that of *r*, i.e. 2*R*_*max*_.

From the obtained representation (6), we then compute similarity transform invariant features using (7) for each value of *l *and *k *[39] with *p *and *q *are used to index these features. Note we reshape *I *into a single row vector of dimensions *D *= *L *× 2*R*_*max *_for later analysis.

(7) $$I\left(p,q\right)=\sum_{k=l}^{k=2R_{\max}}\sum_{m=-l}^{m=l}c_{kl}^{m}\left(c_{kl}^{m}\right)*,p=1...L,q=l...2R_{\max}$$

In order to provide a scalar estimate how different a given thalamus shape was, we calculated the mean of the row vectors, I (Eqn 7) separately for both the left and right thalami. A distance metric, estimating how "abnormal" a given shape was estimated by determining the Euclidean distance between the given feature vector and the mean vector. For example, the distance for right thalamus for the *j*^*th *^subject was estimated as:

(8) $$Dist_{right}^{j}=\sqrt{\sum_{k=1}^{D}(I_{k}^{j^{j}}-I_{right}{)}^{2}}$$

## Competing interests

The author(s) declare that they have no competing interests.

## Authors' contributions

MJM conceptualized the study, supervised the collection of the data from UBC, and supervised the application of the SPHARM technique to medical data. AU developed the SPHARM-based method and performed the calculations. RA supervised the development of the SPHARM-based method and assisted in the application to medical data. SP collected the data at UBC and performed the manual segmentations of the thalami. ML collected the data at UNC and manually segmented the data from UNC. XH supervised the collection of UNC data and assisted in biological interpretation of the results.

## Pre-publication history

The pre-publication history for this paper can be accessed here:


